# Palliative appropriateness criteria: external validation of a new method to evaluate the suitability of palliative radiotherapy fractionation

**DOI:** 10.1007/s00066-022-02040-y

**Published:** 2023-01-10

**Authors:** Carsten Nieder, Ellinor C. Haukland, Bård Mannsåker, Astrid Dalhaug

**Affiliations:** 1 Department of Oncology and Palliative Medicine, Nordland Hospital Trust, 8092 Bodø, Norway; 2 Department of Clinical Medicine, Faculty of Health Sciences, UiT—The Arctic University of Norway, Tromsø, Norway; 3 SHARE—Center for Resilience in Healthcare, Faculty of Health Sciences, Department of Quality and Health Technology, University of Stavanger, Stavanger, Norway

**Keywords:** Radiation therapy, Bone metastases, Brain metastases, Prognostic factors, 30-day mortality

## Abstract

**Background:**

Recently, the palliative appropriateness criteria (PAC) score, a novel metric to aid clinical decision-making between different palliative radiotherapy fractionation regimens, has been developed. It includes baseline parameters including but not limited to performance status. The researchers behind the PAC score analyzed the percent of remaining life (PRL) on treatment. The latter was accomplished by calculating the time between start and finish of palliative radiotherapy (minimum 1 day in case of a single-fraction regimen) and dividing it by overall survival in days from start of radiotherapy. The purpose of the present study was to validate this novel metric.

**Patients and methods:**

The retrospective validation study included 219 patients (287 courses of palliative radiotherapy). The methods were identical to those employed in the score development study. The score was calculated by assigning 1 point each to several factors identified in the original study and using the online calculator provided by the PAC developers.

**Results:**

Median survival was 6 months and death within 30 days from start of radiotherapy was recorded in 13% of courses. PRL on treatment ranged from 1 to 23%, median 8%. Significant associations were confirmed between online-calculated PAC score, observed survival, and risk of death within 30 days from the start of radiotherapy. Patients with score 0 had distinctly better survival than all other groups. The score-predicted median risk of death within 30 days from start of radiotherapy was 22% in our cohort. A statistically significant correlation was found between predicted and observed risk (*p* < 0.001). The original and present study were not perfectly concordant regarding number and type of baseline parameters that should be included when calculating the PAC score.

**Conclusion:**

This study supports the dual strategy of PRL and risk of early death calculation, with results stratified for fractionation regimen, in line with the original PAC score study. When considering multifraction regimens, the PAC score identifies patients who may benefit from shorter courses. Additional work is needed to answer open questions surrounding the underlying components of the score, because the original and validation study were only partially aligned.

## Introduction

Palliative radiotherapy plays an important role in the multimodal management of patients with incurable cancer [1, 2]. Goals of treatment (pain relief, tumor growth inhibition, prolongation of survival) vary and are influenced by several patient- and disease-related factors, e.g., patient preference, performance status (PS), overall tumor burden, availability and efficacy of systemic anticancer treatment, and size of the radiation target volume [3–5]. The recent scientific focus on radiotherapy personalization holds promise with regard to prescription of patient-specific fractionation regimens [6, 7]. The primary aim of many publications was to analyze death within 30 days and to provide predictive tools that may assist clinicians who wish to avoid prolonged fractionation regimens in the final phase of cancer progression [8–11]. On the other hand, efforts towards short-course fractionation should not lead to harm in terms of withholding appropriate, higher-dose radiotherapy in patients surviving long enough to experience benefit. Balancing potential over- and undertreatment and finding the best individual strategy has always been challenging. Many studies have reported rates of death within 30 days that range from 8 to 15%, with considerable interstudy heterogeneity [8–11].

Recently, Farris et al. suggested a pragmatic method to evaluate the suitability of palliative radiotherapy fractionation [12]. They described a novel metric, the palliative appropriateness criteria (PAC) score, after having analyzed the percent of remaining life (PRL). The latter was accomplished by calculating the time between the start and finish of palliative radiotherapy (minimum 1 day in case of a single-fraction regimen) and dividing it by overall survival in days from the start of radiotherapy. Factors significantly associated with a long time spent on treatment, i.e., increased PRL (and therefore included in the PAC score; 1 point each to several factors), were male gender, Eastern Cooperative Oncology Group (ECOG) performance status (PS) 3–4, lung or “other” primary diagnosis (vs. breast or prostate), radiotherapy indication (neurological dysfunction vs. pain/other), inpatient status, and extraosseous site treatment. However, factors were not uniform across all different fraction regimens. For example, only four factors were relevant in the subgroup selected for single-fraction irradiation. Farris et al. provided an online risk assessment tool allowing for calculation of individual patients’ PAC score. Our group was interested in further assessment of this new tool, because it employs readily available information and is not very time consuming. Therefore, we performed an external validation study of the PAC score.

## Materials and methods

This retrospective single-institution study resembled the retrospective single-institution study by Farris et al. to ensure comparability of the two cohorts. In the original study (1027 courses in 850 patients), inclusion was limited to 2014–2018 and 1, 2–5, or 10 fractions [12]. In order to ensure sufficient cohort size, our inclusion time period was extended (2014–2019; 1, 2–5, or 10 fractions; no exclusion of patients who failed to complete all prescribed fractions). Our cohort consisted of 219 consecutive patients (287 courses) managed with standard palliative external beam radiotherapy techniques, excluding stereotactic ablative radiotherapy. Examples include painful bone metastases irradiated with a single fraction of 8 Gy, multiple brain metastases managed with whole-brain radiation (5 fractions of 4 Gy), or symptomatic supraclavicular lymph node metastases (10 fractions of 3 Gy). Fractionation was at the discretion of the treating oncologist and all patients also received standard-of-care systemic anticancer treatment, if indicated and feasible (organ function, comorbidity, PS). For the purpose of quality-of-care monitoring and validation of innovative scores or nomograms, our institution maintains a review board-approved database [13, 14], which was used for the present study.

**Fig. 1:**
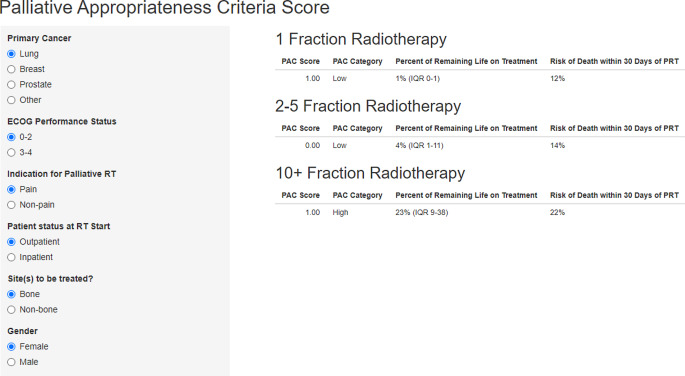
Example (test case): online calculation of the palliative appropriateness criteria (*PAC*) score

The methods were identical to those employed by Farris et al. [12]. Overall survival (time to death) from the first day of radiotherapy was calculated by employing the Kaplan–Meier method for all 287 courses. Different groups were compared using the log-rank test (SPSS 28, IBM Corp., Armonk, NY, USA). Only 27 survival outcomes were censored after a median of 36 months of follow-up (minimum 28 months). Date of death was known after all other courses. Descriptive analyses were performed using count (frequency) and mean (standard deviation). PRL was described using median and compared across groups using the Kruskal–Wallis test. *P*-values < 0.05 were considered statistically significant. The PAC score was calculated by assigning 1 point each to several factors previously identified in the original study, and the online calculator was employed to perform this validation study (https://ryhughes.shinyapps.io/pacs/). Figure 1 shows the screen displaying the results of a test calculation (hypothetical patient).

## Results

The median age was 69 years, range 32–91 years. The largest proportion of courses was administered in patients with prostate or lung cancer and in the outpatient setting, as shown in Table 1. The main indication was painful bone metastasis without simultaneous inclusion of non-bone target volumes. Forty-one percent of courses included 2–5 fractions (intention-to-treat), 35% 10 fractions, and 24% a single fraction. Courses remained incomplete in 3% (*n* = 9). Median overall survival was 6 months (95% confidence interval [CI] 4.5–7.3 [all 287 courses], 1‑year rate 32%, 2‑year rate 17%). Death within 30 days from start of radiotherapy was recorded in 37 of 287 courses (13%). PRL on treatment (time between start and finish of palliative radiotherapy divided by overall survival in days from start of radiotherapy) ranged from 1 to 23%, median 8%.

**Table 1:** Baseline characteristics, *n* = 287 treatment courses

Baseline parameter	Number	Percent
Female sex	118	41
Male sex	169	59
Age ≤ 60 years	62	22
Age 61–70 years	92	32
Age 71–80 years	94	33
Age ≥ 81 years	39	14
Prostate cancer	72	25
Non-small cell lung cancer	56	20
Breast cancer	53	19
Small cell lung cancer	11	4
Renal cell cancer	17	6
Colorectal cancer	32	11
Bladder cancer	10	4
Malignant melanoma	6	2
Other primary tumors	30	10
ECOG PS 0	18	6
ECOG PS 1	93	32
ECOG PS 2	91	32
ECOG PS 3–4	85	30
Outpatient	182	63
Inpatient	105	37
One or two target volumes irradiated	206	72
Three or more target volumes irradiated	81	28
Previous RT (curative or palliative), 1 course	86	30
Previous RT, > 1 course	61	21
No previous RT	140	49
Osseous metastases irradiated (exclusively)	234	82
Extraosseous metastases irradiated	53	18
Pain indication for RT	245	85
Non-pain indication (neurological etc.)	42	15
Prescribed regimen of 10 fractions	100	35
Prescribed regimen of 1 fraction	70	24
Prescribed regimen of 2–5 fractions	117	41
No systemic therapy	63	22
Previous or ongoing systemic therapy	224	78
Corticosteroid concomitant to RT	115	40
No corticosteroid concomitant to RT	172	60
Opioid analgesic concomitant to RT	189	66
No opioid analgesic concomitant to RT	98	34
Palliative care team involved	96	33
Palliative care team not involved	191	67
Early RT, within 2 months from cancer diagnosis	91	32
Late RT, > 2 months	196	68

*ECOG* Eastern Cooperative Oncology Group, *PS* performance status, *RT* radiotherapy

Table 2 displays the association between online-calculated PAC score, observed survival, and risk of death within 30 days from start of radiotherapy. These results confirm the ability of the PAC score to predict the risk of death within 30 days from start of radiotherapy (*p* = 0.002). However, very few patients in our study had high PAC scores, i.e., 4–6. The median score was 1. Patients with score 0 had distinctly better survival than all other groups. The score-predicted median risk of death within 30 days from start of radiotherapy was 22% in our cohort (range 8–39%). As shown in Table 3, a statistically significant correlation was found between predicted and observed risk (*p* < 0.001). However, the agreement was not perfect, in particular in the intermediate-risk group (predicted risk: 22%, observed: 9%), which was relatively large (*n* = 80 courses, minimum 19).

**Table 2:** The palliative appropriateness criteria (PAC) score and observed survival outcomes in 287 treatment courses

Score	Number, percent	Median survival (days)^a^	Death within 30 days from start, percent^b^
0	109, 38	392	4
1	85, 30	107	16
2	56, 20	88	16
3	24,8	74	25
4	9, 3	83	22
5	3, 1	110	33
6	1, 0	29	100

^a^from actuarial Kaplan–Meier curves, *p* < 0.001 (log-rank test, pooled over all seven strata)

^b^*p* = 0.002 (chi-square test)

**Table 3:** Death within 30 days from start of palliative radiotherapy: online calculator prediction versus observed results

Predicted risk, percent	Observed results, percent^a^	Observed results, *n*
8	0	0/19
12	5	2/43
14	5	3/66
22	9	7/80
37	29	15/52
39	37	10/27

^a^*p* < 0.001 (chi-square test)

The individual components of the PAC score were tested for their association with PRL in the largest cohort of our study (*n* = 117 courses, those with 2–5 fractions). We chose to limit this part of the study to two cohorts to avoid problems resulting from low statistical power/small subgroups, and because we felt that two examples would be sufficient to provide data of interest. Farris et al. showed that ECOG PS 3–4 and irradiation of extraosseous sites were the only risk factors in patients treated with 2–5 fractions. In the present study, both of these could be confirmed (ECOG PS 3–4: median 12% versus 4% if PS was 0–2; *p* < 0.001; extraosseous: median 12% versus 4% if bone only; *p* < 0.001). However, inpatient status was significant too (median 12% versus 4% in outpatients; *p* < 0.001). Farris et al. suggested that this parameter was significant in the two other groups, but not in the 2–5-fractions group.

Regarding the second largest cohort (10 fractions, *n* = 100 courses), we were able to confirm 4 out of 5 risk factors, i.e., ECOG PS 3–4, extraosseous site irradiation, male gender, inpatient, and non-pain radiotherapy indication. The last risk factor, lung or “other” primary cancer (vs. breast or prostate), was not significantly associated with PRL. Primary cancer type nevertheless played a role, because breast cancer (median 8%) performed better than all non-breast types combined (median 23%; *p* < 0.001).

## Discussion

This study was performed to provide additional data about the performance of the PAC score in an independent validation cohort. On the one hand, clinicians already have a considerable number of established scores to choose from [10, 11, 15–19]. On the other hand, the PAC score provides attractive features such as an assessment of the risk of death within 30 days from the start of radiotherapy and stratification for three different, clinically relevant fractionation alternatives. Calculation is not very time consuming, and the necessary information is readily available. In the original US study, Farris et al. reported a median time on treatment of 12 days and that 92% of courses were completed as planned [12]. Median age was 64 years, lung cancer present in 38% (largest subgroup), 69% were outpatients, 80% irradiated to just one site, and 19% with a single fraction. Our cohort was smaller, older (median 69 years), included fewer treatments for lung cancer (24%), fewer outpatients (63%), fewer courses with just a single target volume (33%), and a larger proportion of single-fraction treatment (24%). According to Farris et al., median overall survival was 134 days (95% CI 118–153), i.e., 4.4 months (versus 6 in our study), and 15% of patients were treated with so-called futile radiotherapy (died within 30 days of start) compared to 13% in our analysis. PRL on treatment was 6% (Farris et al.) compared to 8%. Overall, these figures were not very different.

Specific factors included in the PAC score varied among treatment regimens, meaning that factors identified for the single-fraction regimen were not the same as those for the 2–5-fraction regimens or the 10-fraction regimens. ECOG PS 3–4 was universally associated with significantly higher PRL among all three regimens. Lung or “other” primary, non-pain radiotherapy indication (e.g., neurological), and inpatient status were associated with higher PRL for 1‑ and 10-fraction regimens. Extraosseous site of palliation was associated with higher PRL for 2–5- and 10-fraction regimens, whereas male gender was only found to be significant for 10-fraction regimens. The present study confirmed most of these factors; however, concordance was not perfect. Despite slight differences, the PAC score predicted the risk of death within 30 days from the start of radiotherapy (*p* = 0.002). However, comparison was hampered by the fact that very few patients in our study had high PAC scores, i.e., 4–6. Possibly, many of these patients were not referred (managed with best supportive care rather than radiotherapy).

Our results appear promising and justify an additional, definitive validation study in a larger cohort of patients. Besides study size, the retrospective single-institution evaluation can be considered the main limitation of the present work. Moreover, fractionation options different from those included in the PAC score studies also exist. Currently, uncertainty exists surrounding the factors that should be part of the PAC score. Additional factors, which may be explored in a larger, future study, include blood test results [11], previous and ongoing systemic therapy, and prior hospitalizations [18].

In a previous study [10], 30-day mortality was highest in inpatients 68 years of age or older (59%) and lowest in outpatients with ECOG PS 0–2 (13%). A total of five different prognostic categories were identified. Another, rather complex predictive scoring system had 10 predictor variables, including but not limited to blood test results [11]. The TEACHH survival prediction model, which did not focus specifically on 30-day mortality, includes cancer type (lung and “other” versus breast and prostate), older age (> 60 years versus ≤ 60 years), liver metastases, ECOG PS (2–4 versus 0–1), hospitalizations within 3 months before palliative radiotherapy (0 versus ≥ 1), and prior palliative chemotherapy courses (≥ 2 versus 0–1) [18]. Other authors have advocated diagnosis-specific and/or irradiated site-specific scores, e.g., for bone and brain metastases [15, 16]. Even simple models such as the one introduced in 2008 by Chow et al. (three factors: non-breast cancer, metastases other than bone, and Karnofsky PS ≤ 60) have demonstrated clinical value [17, 20].

Survival predictions in clinical oncology care tend to be overly optimistic [21]. They sometimes lead physicians to recommend and start palliative radiotherapy (or other treatments) which cannot be completed because of rapid deterioration of the patient’s general condition and/or organ function. In an analysis of patients who died during palliative radiotherapy, Berger et al. found that once radiotherapy had begun, the treatment duration required a median of 64% of the remaining lifetime [22]. It appears unrealistic to achieve perfectly tailored treatment in 100% of patients at the time being, irrespective of the prognostic assessments one chooses to implement. However, minimizing the rates of permanent treatment discontinuation and PRL on treatment are important goals, especially in settings with long waiting lists and limited resources, where futile attempts to palliate symptoms in terminal patients may compromise outcomes in others who have a lot more to gain if timely treatment is possible. The latter group includes, e.g., patients with limited metastatic disease [23, 24].

Even if it remains unclear whether or not the PAC score is able to outperform other scores (head-to-head comparison should be delayed until definitive validation and optimization are completed), the principle of providing separate predictions for a range of fractionation regimens appears highly relevant. Obviously, there were reasons for the treating physicians to prefer short-course treatment rather than 10 fractions in the cases where such fractionation regimens were selected in both PAC score studies. The exact triggers remain unknown (in the present study cohort, none of the other scores was consistently used), but likely a complex interplay of PS, tumor burden, organ function, patient preference, etc. explains the decisions that were made. It appears possible to design a prospective study that compares standard decision-making to PAC (or other) score-based decision-making with the endpoints of PRL on treatment and death within 30 days from start, as well as patient decision regret, palliative efficacy, and quality of life.
